# Is combined topical with intravenous tranexamic acid superior than topical, intravenous tranexamic acid alone and control groups for blood loss controlling after total knee arthroplasty: A meta-analysis: Erratum

**DOI:** 10.1097/MD.0000000000006208

**Published:** 2017-02-17

In the article “Is combined topical with intravenous tranexamic acid superior than topical, intravenous tranexamic acid alone and control groups for blood loss controlling after total knee arthroplasty: A meta-analysis”,^[1]^ which appeared in Volume 95, Issue 51 of *Medicine*, Chunmin Lin's name was originally misspelled in the byline.
